# Same-day discharge after percutaneous coronary procedures—Consensus statement of the working group of interventional cardiology (AGIK) of the Austrian Society of Cardiology

**DOI:** 10.1007/s00508-024-02348-y

**Published:** 2024-05-14

**Authors:** Mathias C. Brandt, Hannes Alber, Rudolf Berger, Ronald K. Binder, Julia Mascherbauer, Alexander Niessner, Martin Schmid, Matthias Frick, the Austrian working group of interventional cardiology

**Affiliations:** 1 Department of Internal Medicine II, Paracelsus Medical University, Müllner Hauptstrasse 48, 5020 Salzburg, Austria; 2 Department of Cardiology, Public Hospital Klagenfurt am Woerthersee, Klagenfurt am Woerthersee, Austria; 3 Department of Internal Medicine, Brothers of Saint John of God Eisenstadt, Eisenstadt, Austria; 4 Department of Cardiology and Intensive Care, Klinikum Wels, Wels, Austria; 5 Department of Internal Medicine 3/Cardiology, University Hospital St. Pölten, Karl Landsteiner University of Health Sciences, Krems, Austria; 6 Division of Cardiology, Department of Internal Medicine II, Medical University of Vienna, Waehringer Guertel 18–20, 1090 Vienna, Austria; 7 Department of Cardiology, Ordensklinikum Linz Elisabethinen, Linz, Austria; 8 Department of Internal Medicine I and Cardiology, Teaching Hospital Feldkirch, Feldkirch, Austria

**Keywords:** Outpatient coronary procedure, Coronary intervention, Ambulatory treatment, Consensus document, Austria

## Abstract

**Introduction:**

Percutaneous coronary intervention is a well-established revascularization strategy for patients with coronary artery disease. Recent technical advances such as radial access, third generation drug-eluting stents and highly effective antiplatelet therapy have substantially improved the safety profile of coronary procedures. Despite several practice guidelines and a clear patient preference of early hospital discharge, the percentage of coronary procedures performed in an outpatient setting in Austria remains low, mostly due to safety concerns.

**Methods:**

The aim of this consensus statement is to provide a practical framework for the safe and effective implementation of coronary outpatient clinics in Austria. Based on a structured literature review and an in-depth analysis of available practice guidelines a consensus statement was developed and peer-reviewed within the working group of interventional cardiology (AGIK) of the Austrian Society of Cardiology.

**Results:**

Based on the available literature same-day discharge coronary procedures show a favorable safety profile with no increase in the risk of major adverse events compared to an overnight stay. This document provides a detailed consensus in various clinical settings. The most important prerequisite for same-day discharge is, however, adequate selection of suitable patients and a structured peri-interventional and postinterventional management plan.

**Conclusion:**

Based on the data analysis this consensus document provides detailed practice guidelines for the safe operation of daycare cathlab programs in Austria.

## Introduction

Percutaneous coronary procedures (PCP), including diagnostic angiography (DA) and percutaneous coronary interventions (PCI), are evolving procedures with vast recent improvements regarding access site technique, interventional tools, and concomitant medical treatment. Consistently, the complication profile of PCPs has substantially improved, showing a decline in access site-related, intraprocedural and postprocedural complications [1, 2]. The periprocedural management of PCP patients, however, has remained mostly unchanged, with the majority of centers in Austria scheduling at least one mandatory overnight stay after procedure. On the other hand, consistent with other European countries, the frequency of PCPs performed in Austria is constantly rising, binding greater in-patient resources and beds for hospitals, and creating higher costs for healthcare providers. Especially in the aftermath of coronavirus disease 2019 (COVID-19) many hospitals are struggling with staff shortages, compromising the operation of elective catheterization laboratory programs.

Medical care for patients undergoing PCPs should reflect the current technical standard of these procedures and the current complication profile. Therefore, considering the procedure’s safety profile the current standard of treating PCP patients as in-patients with at least one overnight stay irrespective of the medical history or procedural complexity is no longer the only possible approach. There are robust data from patients in randomized trials and large observational studies supporting the safety of performing PCPs as same-day discharge (SDD) procedures for selected patients [3–5]. Furthermore, patient preference towards having PCPs performed as SDD procedures is well documented, especially when performed with radial access [6–8]. Therefore, in an approach of shared decision making, patient preference should be taken into consideration when scheduling PCPs as inpatient or SDD procedures. Also, as highlighted by the American College of Cardiology (ACC) [3], 8% of hospitalizations are associated with highly undesirable events as hospital-acquired infections or falls with subsequent injuries [9, 10]. In this context, SDD PCP programs have been initiated by several cardiology departments in Austria not only to provide better service for cardiology patients but also to create a more efficient patient flow around the catheterization laboratories as critical resources.

In Europe as well as in the USA a remarkable heterogeneity of hospital policies with respect to the treatment standard for PCPs and the frequency of SDD PCPs has been documented [11]. Like any other treatment decision, the discharge policy has important consequences for the patient as well as legal implications for the treating physicians. Thus, a consensus on procedural standards reflecting the available scientific evidence that is supported by the Austrian Society of Cardiology is highly desirable. Furthermore, a common consensus incorporating the experience of clinics running SDD PCP programs for selected patients could serve as a valuable reference standard for quality of care.

## Aims

The goals of this consensus document are (1) to provide an overview of the current practice for performing PCPs as an outpatient procedure in Austria and across the world, (2) to review the most recent evidence regarding the safety and feasibility of performing SDD and (3) to provide a consensus framework for the safe and efficient implementation of outpatient clinics within cardiology departments to run a daycare/SDD PCP program. The third goal includes specific recommendations, based on currently available data and experience of the clinics involved, to provide guidance on (4) which patients are best suited for SDD PCP, (5) what intraprocedural techniques or precautions can increase the safety of SDD PCP and (6) what is the appropriate observation period before the patient is discharged as an outpatient.

These recommendations represent the common main aspects endorsed by the working group of interventional cardiology (AGIK) of the Austrian Society of Cardiology and are not intended to be finite or binding. Interventional teams and cardiology departments will make individual modifications to meet their specific expertise and experience. There are different approaches to daycare PCP based on the individual hospital structure and staff training. Furthermore, the authors would like to emphasize that the decision to develop a clinical daycare program around a catheterization laboratory to expand the service offered to patients and referring doctors should be considered an individual choice for every cardiology department. A concept that works well in one hospital could easily be unfeasible in other centers with different local contributing factors. It is not the aim of this consensus document to initiate a competition or create peer pressure as to who is performing the most complex interventions via SDD, but rather to provide guidance on current recommendations and a scientific evidence base for the safe and successful operation of interventional outpatient clinics.

## Methods

### Consensus development

Following the ACC methodology for creating expert consensus decision pathways [12], this consensus document was developed as a draft outline within the working group and circulated among a task force of the AGIK for peer review. Individual practice guidelines from all individual interventional outpatient clinics were integrated. The final document was once again circulated for organizational review and approval from the Austrian Society of Cardiology.

### Review of scientific evidence

This position paper is based on three consensus documents on SDD PCI published by the SCAI [4, 5, 13] and one from the ACC [3]. Furthermore, a systematic literature review and meta-analysis was performed by the Austrian Society of Cardiology focusing on SDD after radial access PCI [14].

## Current trends for PCP in an outpatient setting

### International trends

Reports on PCIs as daycare procedures in selected patients have been published since the late 1980s [15], remarkably in an era where femoral access was still the procedural standard and balloon angioplasty alone the dominant treatment option for coronary artery stenoses. Since then many centers have adopted radial access PCI as the standard treatment option for elective patients and SDD PCI numbers are steadily increasing in the USA and across Europe:

In their 2021 cross-sectional analysis of the American CathPCI Registry Bradley et al. studied the frequency of patients undergoing elective PCI discharged on the day of procedure [16]. From a total of 819,091 elective PCIs in 1716 hospitals, the survey showed a rapid increase of SDD PCI from 4.5% in 2009 to 28.6% in 2017, a trend that was even more prominent in radial access procedures (increase from 9.9% to 39.7%) compared to femoral access PCI (increase from 4.3% to 19.5%). Dividing the study duration into three time intervals (2009–2011, 2012–2014, 2015–2017), the authors also demonstrated a substantial increase in radial access PCI among SDD (21.8%, 43.4% and 58.3%, respectively) and overnight stay patients (7.8%, 20.1% and 32.6%, respectively) [16]. Among SDD PCI procedures, the percentage of proximal LAD and high risk or type C lesion PCIs also significantly increased over the study period. On the other hand, the authors show a large hospital-level variation of SDD frequencies with 25% of the sites studied discharging < 10% of their patients on the day of PCI despite radial access [16].

Similar trends were published in a Canadian study showing data from 35,972 procedures from 17 clinics in and around Ontario, Canada, from 2008 to 2015 [17]. During the study period the total rate of SDD PCI increased from 17% in 2008 to 45% in 2015; however, as in the previous study, a substantial interhospital variation was detected, including 17 clinics with 0–17% SDD PCI. Indicators of high SDD PCI rates were (1) university clinics and university-affiliated teaching hospitals (40.1% vs. 10.7%), (2) presence of onsite cardiac surgery departments (34.9% vs. 9.4%), and (3) centers with > 50% radial access use (42.8% vs. 25.9%) [17].

A 10-year single-center study from France (from 2007 to 2016) showed a comparable increase in SDD PCI procedures from 14% in 2008 to 32% in 2015 [18]. Comparing SDD vs. overnight stay patients, the authors found that in the SDD cohort radial access was used more often, two-vessel CAD was less frequent, and the average number of stents implanted per procedure was lower [18]. The most common reason for overnight admission was the wish to monitor patients in the absence of any complications.

It is worth noting that the increase in SDD PCI procedures runs parallel with a general temporal trend of patients referred to the catheterization laboratory being older, having more comorbidities and a higher calculated risk score for coronary interventions [2]. In a retrospective longitudinal analysis from the Veteran’s Affairs Clinical Assessment, Reporting and Tracking Program data from 2009 to 2015 Waldo et al. detected a significant increase in the proportion of DA and PCI patients who had a high Framingham risk score. The hazard ratio (HR) for mortality, however, remained constant over time, with a nonsignificant decrease among those undergoing intervention (HR 0.983; 95% confidence interval, CI 0.967–1.000). Similar to the trends in SDD procedures cited above, the authors found a steep increase in radial access from 5% to 32% [2].

### Current clinical practice in Austria

A substantial number of Austrian cardiology centers have introduced daycare beds or daycare departments in order to offer a specific scope of procedures on a SDD basis. Generally, DA, PCI, right heart catheterization, cardioversion, loop recorder implant, pacemaker/automatic internal cardioverter defibrillator device replacement as well as administration of intravenous therapy, such as iron infusion, diuretic or antibiotic therapies are being offered as SDD procedures. Depending on the inventories and local environments of the clinics, there are individual policies considering the scope and extent of SDD services. In all centers, local guidelines exist detailing conditions when to switch treatment of a patient back to OS and spare bed capacities are reserved for this purpose.

Currently SDD PCPs are offered by 77% of all cardiology centers in Austria. Of these clinics 40% are running the ambulatory procedures via dedicated outpatient departments and 55% have integrated outpatient beds into regular wards (5% missing data). Fixed protocols or checklists for accepting or rejecting patients for ambulatory procedures have been established in 85% of Austrian cardiology centers. While 77% of clinics offer DA as SDD procedures, only 35% are performing PCI in an outpatient setting. There is some heterogeneity of local protocols with single-stent PCI considered feasible for SDD in all centers performing PCI in an ambulatory setting, while bifurcations, multiple stent and multivessel (MV) PCI are switched to OS in the majority of centers. The volume of SDD cases varies from 2 per week up to 6 per day, whereas the coronavirus disease 2019 (COVID-19) pandemic with subsequent shortages on routine wards has led to an increase in the volume of SDD cases in almost all clinics.

Different clinics have established various local arrangements to integrate a daycare facility into existing cardiology departments. There are two dominant forms of implementation: (1) SDD beds integrated into regular wards where the same doctors, nurses and service staff treat and monitor outpatients, and (2) dedicated outpatient clinics, sometimes shared between different departments (e.g., cardiology combined with gastroenterology, surgery or dermatology), where specific outpatient doctors and nurses are on duty. One benefit of the first form of outpatient clinics is greater flexibility in discharge times, especially considering the waiting period with wound compression after PCI (sometimes up to 6 h). As staff are present on the regular wards virtually 24 h/7 days per week, discharge can take place without any limitation. On the other hand, SDD capacities would be equally compromised in cases of staff shortages or bed reallocation due to COVID-19. Independent outpatient departments (implementation 2) are somewhat more limited in operating hours, on the other hand the general appearance as a non-ward department is preferred by some patients. The staff are also focused on outpatients only and free from many duties connected with generally more immobile patients on regular wards, which could improve the general treatment atmosphere.

## Safety profile and outcome of SDD PCP

### Review of clinical trials and meta-analyses

As stated above, the Austrian Society of Cardiology has performed a systematic literature review and meta-analysis to substantiate its practice recommendations [14]. In short, four large meta-analyses have published outcome data for SDD PCI [19–22], summarizing 38,785 patients with SDD and 256,049 patients staying overnight. All four meta-analyses are consistent in demonstrating no added risk of SDD practice assessed by MACE at 24 h and/or 30 days.

It could be argued that in all trials including RCTs patients deemed suitable for SDD represent a selected patient population, generally being younger, with a lower burden of chronic medical conditions, less severe degree of CAD and residence not far from the interventional clinic; however, it is the very same selection process that is unanimously being recommended by current advisories to identify potential patients suitable for SDD PCP [3, 4]. It was never intended to offer SDD PCPs to all patients with an indication for coronary angiography or PCI. Conversely, the application of current SDD selection criteria has been shown to be effective: A comparison between randomized and non-randomized (excluded) patients from the RCT by Bertrand et al. [23] showed that the rate of MACE (death, MI, TVR) in patients excluded from randomization was significantly higher at 30 days (10.2% vs. 1.6%), 6 months (17.5% vs. 5.6%), and 12 months (24.5% vs. 9%) as compared with randomized patients [13, 23].

Finally, it should be pointed out that none of the observational studies, RCTs or meta-analyses discussed above featured a protocol where only DA was considered suitable for SDD or where PCI/stent implantation was by default an exclusion criterion for SDD. The safety and outcome data extracted exclusively represent PCI on a SDD basis.

### Data on safety of SDD in specific patients and settings

With growing experience of SDD PCPs and especially PCIs, some centers have extended the SDD concept to more challenging PCI indications, once again highlighting the robust safety profile of this treatment approach [24–29]. We have reviewed the evidence base for SDD PCI in higher-risk settings. This includes complex PCI, elderly patients (age > 75 years), rotational atherectomy, chronic total occlusion PCI, and left main PCI. Briefly, currently available data from a multitude of trials shows no additional risk for these challenging settings. It must be acknowledged, however, that a large number of trials are retrospective analyses from registries and that a significant degree of heterogeneity exists considering the frequency of higher-risk PCIs on a SDD basis.

### Patient satisfaction

Patient preference should be taken into account when scheduling invasive procedures. There is a well-documented patient preference towards radial access for PCP, which leads to less discomfort, less frequent hematomas and better quality of life postprocedure [30, 31].

Across different countries, different study settings and decades of interventional experience SDD after PCP has been shown to be the preferred treatment mode as opposed to OS [6–8, 32–36], satisfying another clear patient preference for short hospital stay, earlier and easier ambulation.

### Cost effectiveness

Numerous international studies on cost efficacy of SDD departments have demonstrated a potential for significant cost savings when performing PCPs in an ambulatory setting, while maintaining the existing catheterization laboratory inventories and staff at the center [35–38].

Data from Austria confirm that for the individual cardiology departments, the setup of an interventional outpatient clinic into an existing infrastructure does not involve large investments. A calculation from the University Clinic Innsbruck has shown that after implementation of a cardiology outpatient clinic, estimated annual treatment costs could be reduced by about 560,000 €, the largest component being cost for personnel (University Clinic for Internal Medicine III, Medical University of Innsbruck; unpublished data), consistent with reductions in fixed hospital costs reported before [37].

## Guidelines and international consensus statements

The concept of SDD PCI is being adopted more frequently in cardiology centers around the world and several cardiology societies have developed consensus documents outlining practice guidelines for a safe and effective performance of PCI via outpatient departments. Table 1 shows a synopsis of (1) the Society for Cardiovascular Angiography and Intervention (SCAI) 2009 expert consensus document focusing on length of stay post-SDD PCI [13], (2) the SCAI update from 2018 [5], (3) the SCAI 2020 update position statement for ambulatory surgical centers [4] and (4) the 2021 American College of Cardiology (ACC) expert consensus decision pathway for SDD PCI [3]. On the far right is (5) the corresponding equivalent for each category from the present Austrian Society of Cardiology consensus.

**Table 1:** Synopsis—Patient or procedure characteristics unfavorable for SDD

	SCAI Expert Consensus Document	SCAI Expert Consensus Update	SCAI Position Statement 2020 for ASC	ACC Expert Consensus Decision Pathway	Austrian Society of Cardiology Consensus
Publication	Chambers et al. (2009) [13]	Seto et al. (2018) [5]	Box et al. (2020) [4]	Rao et al. (2021) [3]	(This publication)
–	Exclusion criteria defined by each consensus/position paper grouped by category				
*Patient*					
Age	Age > 70 years	Age: no limit	Age: no limit	Age: no limit	Age: no limit
Clinical setting	NSTEMI or STEMI	NSTEMI or STEMI	ACS	NSTEMI or STEMI	Staged PCI for NSTEMI or STEMI no exclusion
	Elevation of cardiac biomarkers	Continuing angina		Staged PCI for NSTEMI or STEMI no exclusion	
Clinical condition	Unstable patient	Not clinically stable	Any signs of clinical instability	Any disease exacerbation of COPD/HF/hypertension	Any signs of clinical instability
Mental state	<NS>	<NS>	<NS>	Changes in mental state	Patient is mentally incapacitated
Diabetes	Insulin-dependent diabetes mellitus	Diabetes mellitus not clinically stable	<NS>	Diabetes mellitus: no concern if clinically stable	Diabetes mellitus: no concern if clinically stable especially no risk of hypoglycemia
COPD	COPD if significant or requiring medication	COPD if not clinically stable	Advanced COPD requiring oxygen	COPD: no concern if clinically stable	COPD: no concern if clinically stable
			Severe pulmonary hypertension		
Heart failure	LVEF < 55%	Decompensated HF, fluid overload (No LVEF cut-off)	Decompensated HF (NYHA classes 3 and 4)	HF: no concern if clinically stable (No LVEF cut-off)	HF: no concern if clinically stable (No LVEF cut-off)
			LVEF < 30%		
Valvular heart disease	Any valvular regurgitation	<NS>	Severe aortic stenosis	<NS>	<NS>
Renal function	eGFR < 60 ml/min	CKD not clinically stable	CKD with eGFR < 45 ml/min/1.73 m^2^ BSA	CKD: no concern if clinically stable	CKD no concern if clinically stable
	On dialysis	CKD requiring prolonged hydration			
Peripheral vascular disease (PVD)	Symptomatic PVD	PVD not clinically stable	Significant PVD limiting femoral or radial access	<NS>	PVD not clinically stable
					PVD limiting radial/ulnar access
Cerebro-vascular disease	<NS>	<NS>	Recent TIA or stroke	<NS>	<NS>
Contrast allergy	Any contrast allergy	Contrast reaction with ongoing symptoms	Severe contrast allergy	<NS>	Contrast reaction with ongoing symptoms
Anemia	<NS>	<NS>	Anemia with Hb < 9 g/dl	<NS>	<NS>
Coagulation	<NS>	<NS>	Coagulopathy with INR > 1.5 or platelet count < 100,000	<NS>	<NS>
Social factors	No adequate home support	No caregiver for 24 h postprocedure	No caregiver for 24 h postprocedure	No reliable person for transit home	No adequate home support
			No adult present for discharge and at home	No caregiver for 24 h postprocedure	
Distance to PCI center	Lives or stays > 20 miles from PCI facility	No transportation home	<NS>	Inadequate home support	<NS>
	No adequate home support	Inadequate home support			
Access to emergency medical care/service	Inadequate local emergency medical care	Inadequate access to emergency medical care	≥ 30 min drive time to hospital capable of providing emergency medical care	No access to emergency services	≥ 30 min drive time to hospital capable of providing emergency medical care
*Procedure*					
Access site	Femoral access no exclusion with closure device	Brachial access	<NS>	Femoral access no exclusion with closure device	Femoral access no exclusion with closure device
	Brachial no exclusion				
Sheath	<NS>	Sheath size ≥ 9 French	<NS>	Sheath size > 7 French	<NS>
				Sheathless guide 6.5 French no exclusion	
PCI location	1‑vessel disease with LM PCI	Last remaining artery	Unprotected LM	No exclusions on location (including LM)	No exclusions on location
	Prox. LAD	No exclusion if LM or bifurcation	Bifurcation with significant side branch involvement	Bifurcation no exclusion	
	Any bifurcation		Bypass graft		
	SVG		Last remaining conduit		
	IMA		Extreme prox. angulation/tortuosity		
Multivessel	> 1 vessel PCI	No limit on number of vessels	3‑vessel CAD	No limit on number of vessels	No limit on number of vessels
Stent length/number	> 1 Stent	No limit on stent number	No limit on stent number	No limit on stent number	No limit on stent number
	> 28 mm	No limit on cumulative stent length	No limit on cumulative stent length	No limit on cumulative stent length	No limit on cumulative stent length
Periprocedural adverse events	<NS>	Periprocedural MI	Any cardiac or non-cardiac instability during PCI	Any periprocedural complications	Any cardiac or non-cardiac instability during PCI
PCI success	Balloon angioplasty without stent	Balloon angioplasty without stent	<NS>	Unsuccessful stent deployment	<NS>
		Inability to deliver stent			
Residual stenosis	<NS>	<NS>	Residual stenosis > 30%	Residual stenosis > 50%	<NS>
TIMI flow	<NS>	<NS>	TIMI flow < Grade 3	TIMI flow < Grade 3	TIMI flow < Grade 3
			Transient vessel closure		Transient vessel closure
Side branches (SB)	Any SB loss	Any SB closure	Significant SB involvement	SB: no diameter limit	Any SB loss if clinically significant (ST elevation, persistent AP, arrhythmia)
	Compromised SB flow		SB loss > 1 mm		
Dissection	Any dissection	Any dissection	Types B–F dissection in target vessel at the end of procedure	<NS>	Any dissection not covered by DES or treated with DEB
Thrombotic events	Distal embolization	<NS>	Any intracoronary thrombus	<NS>	Any intracoronary thrombus
CTO-PCI	CTO attempt	CTO attempt	Any CTO	CTO no exclusion if clinically stable	CTO no exclusion if clinically stable
Rotational atherectomy	Any rotational atherectomy	Any rotational atherectomy	Any rotational atherectomy	Rotational atherectomy no exclusion	Calcium modifying therapy no exclusion if clinically stable
Left-ventricular assist device (LVAD)	<NS>	Any LVAD	<NS>	<NS>	Any LVAD
					Any circulatory support (inotropes etc.)
Anticoagulation	Use of GPIIb/IIIa inhibitors	Use of GPIIb/IIIa inhibitors	<NS>	<NS>	<NS>
Contrast medium	Large volume of contrast medium (> 500 ml)	Large contrast volume	<NS>	<NS>	Large volume of contrast medium (> 500 ml)
*Post-procedure*					
Access site	Hematoma	Bleeding complications	Access site hematoma	Bleeding	Clinically relevant bleeding or hematoma
				Vascular complications	
ECG	Any rhythm disorders	(Postprocedure ECG “if ordered”)	ECG abnormalities or rhythm disorder prior to discharge	Persistent ischemic ECG changes	Dynamic ECG changes
				Dysrhythmia	Dysrhythmia
Pain	Continuing chest pain	Continuing angina	Chest pain	Unresolved/severe chest pain	Persistent chest pain
Interventionalist, nursing staff	<NS>	Discomfort of caregiver/physician about SDD	Operator judgement favors OS	<NS>	Discomfort of caregiver/physician about SDD
Patient’s decision	Patient and family not willing to consider early discharge	Discomfort of patient about SDD	<NS>	Patient not willing to be discharged	Discomfort of patient about SDD
*Discharge recommendations*					
Clinical follow-up	Involve quality improvement committee, assess complications, patient satisfaction	Follow-up appointment	Follow-up appointment within 1–2 weeks	Schedule contact call 1 day after discharge	According to local expertise
	Interaction with patient and family			Referral to cardiac rehabilitation	

*ACS* acute coronary syndrome, *CKD* chronic kidney disease, *COPD* chronic obstructive pulmonary disease, *CTO* chronic total occlusion, *DEB* drug-eluting balloon, *eGFR* estimated glomerular filtration rate, *HF* heart failure, *LM* left main coronary artery, *LVAD* left ventricular assist device, *LVEF* left ventricular ejection fraction, *MI* myocardial infarction, *<NS>* not specified, *NSTEMI* non ST-elevated myocardial infarction, *PCI* percutaneous coronary intervention, *PVD* peripheral vascular disease, *SB* side branch, *STEMI* ST-elevated myocardial infarction, SVG saphenous vein graft, *TIA* transitory ischemic attack, *TIMI* thrombolysis in myocardial infarction classification

Comparing exclusion criteria against SDD extracted from the four different sources shows that the 2009 SCAI consensus, being the earliest, is the strictest among all five. Potential patients have to be almost free from any chronic disease irrespective of clinical stability. PCI is limited to a single DES of maximally 28 mm length. Proximal LAD, bifurcations, multivessel PCI (MV) are excluded [13]. The updates from 2018, 2020 and 2021 take into account newer trial results supporting a very favorable safety profile of SDD PCI, therefore widening the range of acceptable patient comorbidities and procedure complexities. Chronic conditions like diabetes mellitus, COPD, chronic kidney disease or heart failure are acceptable if under adequate treatment and clinically stable [3–5]. Instead of defining individual exclusion criteria regarding number of stents, cumulative stent length or number of vessels treated, the newer recommendations emphasize the importance of a complication-free procedural outcome and stable clinical condition of the patient relative to the baseline level preprocedure. Even challenging PCI settings (MV, LM, RA, CTO) are not categorically excluded as long as no periprocedural complications occurred and no exacerbation of any underlying disease of the patient was detected [3]. On the other hand, even in a complication-free postinterventional setting, the patient’s willingness to be discharged on the same day, to understand postinterventional DAPT and to be accompanied by a caregiver at home are crucial criteria before proceeding to discharge.

While this consensus endorses many criteria introduced by the 2021 ACC publication, some decisions require further comment:

Age:

It does not seem practicable to introduce a formal age limit as the individual fitness and coping with provisions necessary to organize a SDD treatment may vary substantially despite the numerical age.

Social factors:

The term “reliable person for transit home” does also include a commercial transport service as long as a qualified caregiver is ready and waiting at the patient’s home residence.

Access site:

With respect to the magnitude of clinical trials confirming the safety of SDD after femoral access PCI [6, 20], this approach is considered safe if vascular closure devices are used and can be implanted without complications. If, however, the access site has to be switched from radial to femoral due to complications, resulting in two compromised body areas, the patient should be rescheduled to OS. This is further supported by data indicating that femoral access is associated with a higher complication rate when used as a secondary access site [39].

PCI location:

While no exclusion criteria are defined based on stent location, LM and bifurcation PCI should match the interventional team’s expertise. Consistent with current guidelines, especially in LM PCI intravascular imaging techniques is mandatory to document proper stent apposition and absence of stent edge dissections.

Cumulative stent length:

Consistent with the ACC 2021 consensus [3], the introduction of a limit on number of stents or vessels treated in SDD PCIs does not seem to be warranted. A multitude of studies in this review have shown no added risk for complex interventions including MV and multistent PCIs. Signalling extensive atherosclerotic disease, cumulative stent length does have an adverse effect on short-term and long-term MACE in BMS [40] and 1st generation DES [41]. In 2nd and 3rd generation DES this correlation has been shown to be either absent [42, 43], or only affecting late outcomes in women [44].

Dissections:

Dissections not treated with DES or DEB should be considered an exclusion criterion for SDD post-PCI. If OCT is being used, this criterion has to be balanced with the fact that small edge dissections are found in up to 40% of OCTs post-DES implant [45]. Of these 80% are not detectable via angiography [46] and have no impact on clinical outcome [47]. Therefore, consistent with current OCT guidelines, clinically relevant edge dissections post-DES implant are defined as either (1) > 200 µm in depth into the vessel wall (media), and/or (2) reference luminal area < 4.5 mm^2^ at either proximal or distal stent edge, and/or (3) ≥ 3 mm in length from the stent edge, and/or (4) spanning > 60° in arc from the center of the vessel [47, 48].

Acute kidney injury (AKI)/contrast-induced nephropathy (CIN):

In CIN serum creatinine levels tend to rise at the end of a 24 h window postcontrast exposure, reaching a maximum at 48–72 h [49]. Therefore, it is very unlikely that CIN could be detected within the 3–6 h postprocedural observation period even with serial creatinine laboratory tests. For this reason, the cut-off for contrast use mandating a switch from SDD to OS strategy should be set at 500 ml as in [13]. A more conservative, dynamic threshold of 3 × calculated eGFR (Cockroft-Gault formula), which accounts for the elevated risk for CIN in patients with renal failure [50] can be considered in individual patients at high risk for CIN.

## Practical recommendations

### Hospital environment

All SDD PCPs should be performed by clinics and medical centers with a high annual workload of coronary procedures. Consistent with the 2020 SCAI recommendations for ambulatory surgical centers, we recommend that only operators with an expertise in interventional cardiology, a personal experience beyond a total of 500 procedures and an annual workload of > 50 procedures should be performing PCPs in the outpatient setting [4]. A specialized hospital program or SOP for SDD PCPs should be in place including secretaries, nurses and catheterization laboratory personnel, defining the relevant steps from assessment of referrals, scheduling of appointments for outpatient evaluation, daycare admission, cathlab procedure, postprocedure patient care, discharge criteria checklist, and clinical follow-up protocol.

At any time from referral to discharge, including the patient’s own condition as well as observations by all levels of personnel, the goal of SDD should be critically re-evaluated.

An experienced colleague should be available at all times to assist in the preprocedural and postprocedural care and to manage discharge formalities for interventional daycare patients. If these tasks are simply added to the list of responsibilities for the interventionalists in the catheterization laboratory, a lack of patient supervision and delays in the discharge process, especially in high volume interventional centers, will be the consequence.

In the outpatient clinic either beds or lounge chairs enabling the patients to relax and enjoy some privacy should be present. While live telemetry of ECG and oxygen saturation as in intermediate care units is not considered necessary, nurses should be present at all times during opening hours and there should be a standard protocol detailing the frequency for preinterventional and postinterventional surveillance. Emergency medical equipment for intubation/resuscitation, pericardiocentesis, and at least one echocardiography device and one ECG printer should be present in the outpatient clinic to facilitate swift diagnostics and intervention if a patient’s condition should deteriorate. If the outpatient beds are included into the inventory of a regular ward, the facilities and technical outfit of the normal ward are sufficient.

### Preprocedural considerations

The plan to perform a coronary procedure in an outpatient setting should be discussed with the patient as early as possible and personal concerns and questions should be addressed in an effort of shared decision making.

Relevant social and medical patient characteristics preventing the planned procedure from being performed in a SDD setting are summarized as a checklist in Table 2. One of the critical criteria at this time is the presence of a partner or relative who could assist in transporting the patient to the clinic and back home for the preclinical outpatient assessment and on the day of the procedure. Furthermore, advanced age or serious medical conditions should be taken into consideration. These include decompensated heart failure with LVEF < 30%, severe kidney disease and uncontrolled diabetes mellitus. If clinically stable and compensated, however, these chronic conditions do not exclude SDD PCP.

**Table 2:** Checklist for SDD evaluation—Unfavorable factors before admission (adapted from [3–5])

*Social factors*	
1.	No adequate home support after the planned procedure
2.	Patient or caregiver not able to reach emergency medical support if necessary
3.	Patient is mentally incapacitated
4.	Language barrier compromising comprehension of instructions
*Medical history*	
1.	LVEF < 30% or decompensated heart failure (NYHA class 3–4)
2.	Decompensated kidney disease (eGFR < 30 ml/min/1.73 m^2^ BSA)
3.	Uncontrolled diabetes mellitus, risk of hypoglycemia
4.	Uncontrolled hypertension (systolic BP > 160 mm Hg despite 3 medications)
5.	Uncontrolled/exacerbated COPD with home oxygen therapy
6.	Severe peripheral artery disease compromising radial/femoral access
7.	Severe contrast dye allergy
8.	Acute myocardial ischemia (ACS, NSTEMI, STEMI), same day transfer to other hospital is possible

*ACS* acute coronary syndrome, *BP* blood pressure, *BSA* body surface area, *COPD* chronic obstructive lung disease, *eGFR* estimated glomerular filtration rate, *LVEF* left ventricular ejection fraction, *NSTEMI* non ST-elevated myocardial infarction, *NYHA* New York Heart Association, *STEMI* ST-elevated myocardial infarction

Checklist to assess feasibility of SDD strategy depending on patient’s social background and past medical history

### Coronary angiography and PCI

Specific early slots in the catheterization laboratory program are required for SDD patients, so that the mandatory period of 3–6 h radial compression and clinical observation can be completed without compromising discharge in the afternoon/evening.

All SDD coronary procedures should only be performed by experienced operators with an expertise in non-femoral puncture techniques including radial or ulnar access. Radial access facilitates early mobilization and is associated with lower mortality, fewer adverse cardiovascular events, and bleeding complications [51, 52]. Furthermore, there is a clear patient preference of radial versus femoral access PCPs [8, 30] (see above). The interventional team should be experienced in treating challenging radial anatomies and potential access site complications. If the access site needs to be switched to brachial or femoral due to vascular calcifications or complications during radial puncture, the patient should be scheduled for OS.

As highlighted by the 2021 ACC [3] and the 2018 SCAI [5] expert consensus documents, the clinical condition of the patient during and postprocedure has a greater impact on the final decision on SDD or the length of stay than individual features of the intervention. As summarized in our literature review [14], there is a wide range of RCTs and observational studies supporting the safety of SDD post-PCI even in challenging settings. Consistent with the ACC and SCAI recommendations, we consider SDD postelective PCI feasible and safe, including multivessel DES implantation, bifurcations, proximal/ostial lesions, and successful CTO. If complications or periprocedural adverse events summarized as a checklist in Table 3 occur, SDD is no longer safely possible and the clinical management should be altered to OS, potentially with constant surveillance and treatment depending on the nature of the adverse event. It should be emphasized that the scope of interventions offered within a daycare clinic should reflect the individual interventional center’s expertise and scope of interventions.

**Table 3:** Checklist—Procedural factors against SDD (adapted from [3–5])

*PCI—Local factors*	
1.	Complication at access site (bleeding, major dissection)
2.	Contrast dye usage exceeding 500 ml [50]
*PCI—Coronary anatomy and procedural factors*	
1.	Complications with coronary artery dissection or perforation
2.	Persistent slow flow, no reflow in target vessel (TIMI < Grade 3)
3.	Thrombus formation in any coronary vessel
4.	Any SB loss if clinically significant (ST-segment elevation, persistent AP, arrhythmia)
5.	Failure to deliver stent post predilatation
6.	Peri-interventional ischemia with ST-segment elevation
7.	Hemodynamic instability with inotropic support or left ventricular assist device
8.	Rhythmologic instability with higher degree AV block or ventricular tachycardia
9.	Highly complex or prolonged procedure that may put the patient at increased risk of adverse outcomes according to the clinical judgement of the operator
*General clinical condition*	
1.	Pulmonary edema requiring oxygen support or diuretic therapy
2.	Persistent ST segment alteration or chest pain
3.	Severe contrast allergy requiring medical therapy
*NO contraindication against SDD policy *[3, 5]	
1.	Single or multivessel PCI including proximal LAD or bifurcation
2.	Multiple DES implants into one or more target vessels irrespective of stent number or cumulative stent length
3.	Overstenting side branch > 1 mm with TIMI 3 flow
4.	Uncomplicated successful CTO attempt
5.	Ulnar access or distal radial access
6.	Staged procedures post initial NSTEMI or STEMI

Intraprocedural and postprocedural adverse events based on local factors, interventional course of events and general medical conditions which are contra-indications against a SDD strategy. The last 6 items are listed to point out specific items compatible with same-day discharge, provided the clinical surveillance from procedure to discharge is without major adverse events.

*CTO* chronic total occlusion, *LAD* left anterior descending coronary artery, *NSTEMI* non ST-elevated myocardial infarction, *PCI* percutaneous coronary intervention, *STEMI* ST-elevated myocardial infarction, *TIMI* thrombolysis in myocardial infarction

A large observational study on SDD PCI from 2012 showed excellent safety data in a broad range of patients outside the SCAI/ACC recommendations [13] at that time (2009). These results highlight that any guidelines or position statements should acknowledge further trends in clinical practice standards with developing clinical evidence on safety.

### Postprocedural monitoring

Consistent with elective DAs and PCIs in OS patients, postprocedural ECGs can be considered, cardiac enzyme tests are not necessary in the SDD setting, except in the presence of periprocedural complications, persistent symptoms indicative of myocardial ischemia or cardiac decompensation. In this context it should be noted that two large retrospective studies with cohorts > 5000 patients have established that while a preinterventional elevation in serum Trop-T correlates with an adverse outcome, in the absence of periprocedural myocardial infarction a postinterventional rise in Trop‑T does not offer any prognostic significance beyond the preinterventional value [53, 54]. In the SDD setting, an age-matched study of 149 SDD versus 154 OS patients showed that post-PCI Trop‑T levels > 5 × upper refererence level (URL) did not indicate any incremental short-term or long-term risk [55]. There is no need for bed rest or confinement to a single room in the daycare clinic. Nevertheless, a standardized monitoring protocol should be in place during the 3–6 h of compression bandage. The puncture site and blood pressure should be checked and recorded at regular intervals (e.g. 1×/h).

In the presence of adverse clinical events, summarized in Table 4, adequate diagnostic measures including BP testing, trans-thoracic echocardiography (TTE), ECG and laboratory tests must be taken. In cases of adverse outcome, the clinical strategy must be changed from SDD to OS and this explained to the patient. This also involves unforeseen social patient factors, e.g., if the personal contact providing transfer from the clinic and home support is no longer available or if the patient may no longer feel comfortable with the concept of SDD.

**Table 4:** Checklist—Postprocedural/clinical observation factors against SDD (adapted from [3–5])

Do NOT schedule SDD if:	
*Procedure outcome*	
1.	Procedural outcome not compatible with SDD (see Table 3)
2.	Any major concern from the interventional team
*Postprocedural monitoring*	
1.	Any clinically relevant decompensation of previous medical conditions (COPD, diabetes mellitus, hypertension, heart failure, renal failure, chronic pain)
2.	Any change in mental state indicative of ischemia or dementia
3.	Persistent chest pain
4.	Persistent ECG abnormalities from baseline ECG
5.	Persistent symptoms of contrast dye allergy
6.	Major hematoma postpuncture site dressing removal
7.	Patient not feeling well, unwilling to be discharged before 24 h observation
*Social factors*	
1.	No contact person for transport home or inadequate home support
2.	Inadequate access to emergency medical care (> 30 min driving time)
3.	Patient not able to take DAPT responsibly

*COPD* chronic obstructive pulmonary disease, *ECG* electrocardiogram, *DAPT* dual antiplatelet therapy, *SDD* same-day discharge

Procedural factors, postprocedural monitoring and social factors incompatible with SDD strategy

### Discharge decision

The final decision to discharge the patient on the same day should finally be made in agreement with the patient and by nurses, catheterization laboratory personnel, interventionalists and an experienced physician at the ward.

If the outcome of the PCP is successful and postinterventional monitoring was uneventful without changes in pre-existing medical conditions, discharge can be scheduled after removal of the radial pressure bandage. The compression time should be 3 h after DA and 4–6 h after PCI. An experienced doctor, ideally the interventionalist, should inspect the patient’s condition and the puncture site after removal of the pressure bandage and give the final decision of discharge.

A structured discharge process with a checklist containing all necessary steps is recommended. An example of a discharge checklist is provided in Table 5. The patient needs to be instructed on changes in the medical treatment, especially on the importance of DAPT in case of PCI with DES implantation. After PCI, the patient should leave the daycare clinic with a prescription of DAPT to provide a gapless concomitant therapy poststent implant. Ideally, a telephone follow-up call is arranged for the next working day in order to verify the patient’s well-being after discharge.

**Table 5:** Discharge checklist (adapted from [5])

Checklist for discharge	
1.	Check access site/radial pulse and cover with sterile dressing
2.	Provide additional wound dressings for ambulatory period
3.	Explain procedure result, changes in medication Document instructions on type and duration of DAPT, if PCI was performed Provide prescription of ASS and P2Y12 inhibitor for 30 days
4.	Explain rules of behaviour to protect the puncture site for 7 days
5.	Provide discharge letter
6.	Instruct the patient on emergency medical service, hand out emergency telephone contact to clinic in case of bleeding, chest pain or other adverse events
7.	Notify the patient of a telephone follow-up call on the following working day

*ASS* acetyl-salicylic acid, *DAPT* dual antiplatelet therapy, *PCI* percutaneous coronary intervention

Concise checklist of clinical assessments and duties to be performed by attending nurses and doctors before discharge from the outpatient clinic

### Clinical follow-up of SDD PCP patients

Consistent with the ACC 2021 expert consensus [3], a clinical follow-up (FU) of SDD PCP patients should be considered in order to collect data on perceived quality of care, potential complications post discharge and to reinforce DAPT medication adherence. This could be done via telephone call on the day post PCP, without the patient having to visit the outpatient department again. It has been documented in prospective studies that DAPT adherence post-SDD PCI is high (87–95%) [8, 56]. In the latter study, nurse-led post-SDD PCI telephone interviews had no influence on P2Y12 inhibitor adherence (telephone 95% vs. control 93%, *p* = 0.627) [56]; however, the authors did find that routine follow-up calls significantly reduced the frequency of unscheduled patient readmittance (8% vs. 16%, *p* = 0.048), as well as self-initiated contacts to general practitioners (29% vs. 42%, *p* = 0.02) [56]. A study from the Netherlands formally assessed the preferred mode of FU post-SDD treatment in 1797 patients treated from 2008 to 2012 [57]. Remarkably, the majority of patients (69.9%) preferred a FU by mail questionnaire, while only a smaller portion of patients preferred a telephone call (13.4%) or email (12.7%; *p* < 0.001) [57]. It is open to speculation, if this trend has changed in the meantime with a more dominant role of mobile telephones and email access even for older patients. While the AGIK does not mandate strict clinical follow-ups as they bind substantial resources, it must be pointed out that due to the novelty of the concept of SDD and the substantial impact it has on the post-PCI FU process, individual FU data from Austria would be valuable. Alternatively, patient follow-up and aftercare could be performed by resident physicians if close cooperation with the team of the catheter lab is guaranteed. The more critical an individual interventional center views the concept of SDD, the more rigorously it should be collecting clinical FU data.

## Conclusion

Due to the rapid evolution of PCPs with predominantly radial access, 3rd generation DES and modern concomitant medical treatment including P2Y12 inhibitors, the safety profile has substantially improved. As discussed above, large longitudinal studies have documented an increase in radial access PCI and a stable low risk for MACE during/post-PCPs despite a trend towards more elderly, more compromized patients and more complex procedures [2]. Despite more recent trials enrolling larger numbers of complex patients [28] and procedures like MV, multistent, bifurcation, CTO [58] and LM PCIs [59, 60], 24‑h and 30-day MACE frequencies have remained at low rates comparable to OS patients. Gilchrist et al. performed 100 SDD versus 665 OS PCIs in higher risk patients including complex and MV PCI [61]. Although only 15% of ambulatory patients would have qualified for SDD according to the 2009 SCAI criteria [13], the authors showed excellent safety data with absence of any MACE from 6–24 h or at 30 days. These findings highlight the rapid evolution of clinical practice surrounding SDD catheterization laboratory programs.

Austrian interventional cardiology clinics have widely adopted this concept and offer PCPs on a SDD basis. Interventional outpatient departments require experienced personnel at all levels familiar with proper patient stratification preprocedure, PCI via radial access even in complex cases and structured surveillance postprocedure. The Austrian Society of Cardiology has considered recent publications from the ACC [3] and SCAI [4, 5, 13] and a wide scope of publications on radial access SDD PCI to develop an evidence base for its own individual position. Based on data from 4 expert consensus statements [3–5, 13], 4 large meta-analyses [19–22], and a separate literature review performed by the AGIK [14], spanning 21 years of clinical evidence from 2001 to 2022, the practice of SDD DA and PCI can be considered safe and feasible for a subset of CAD patients carefully selected by the criteria detailed above (Table 2). The SDD outpatient clinics provide additional value in fulfilling a well-documented patient preference towards shorter hospital stay following PCPs. Furthermore, SDD has been shown to provide substantial cost-saving potential.

**Fig. 1:**
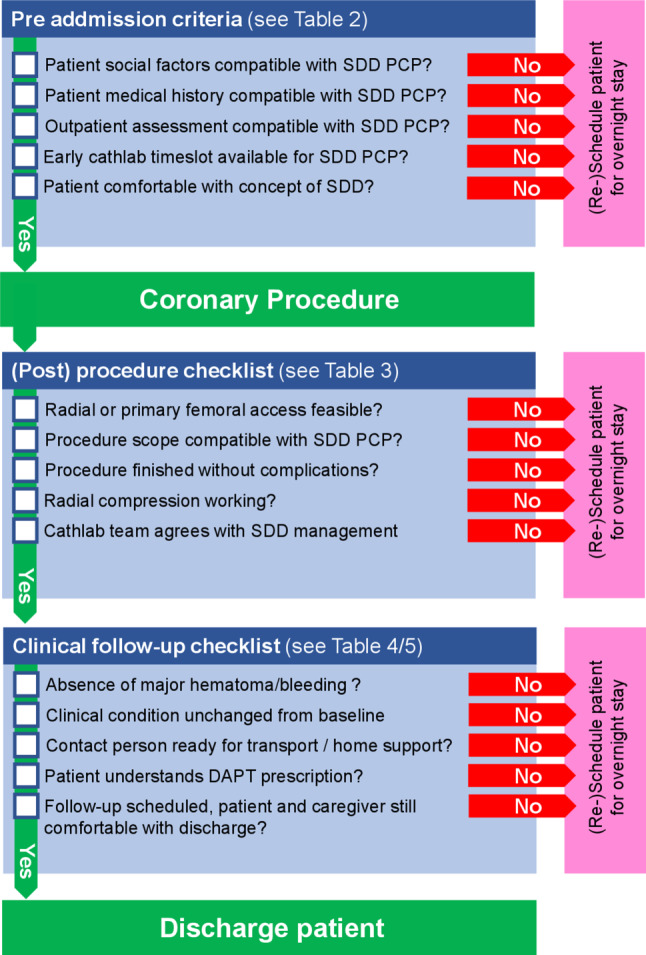
Concise checklist summarizing inclusion/exclusion criteria for SDD strategy prior to admission, during/after coronary procedure and during clinical follow-up/before discharge

Three principles seem to be of paramount importance for a successful outcome: (1) adequate patient selection with clinical assessment by an experienced cardiologist and (2) adjusting the complexity and scope of SDD procedures to the general experience and scope of the interventional team. (3) Adequate aftercare supplied by experienced medical personnel in or out of hospital should be guaranteed. A concise checklist summarizing pre admission, procedural, post-procedural, and follow-up criteria is illustrated in Fig. 1.

These recommendations are subject to changes with further publications in this area and not intended as finite or binding. The interventional centers’ individual policies and interventionalists’ expert opinions are the most important domains in transforming these recommendations into daily clinical practice.

## Abbreviations

ACS: Acute coronary syndrome
AKI: Acute kidney injury
CAD: Coronary artery disease
CIN: Contrast-induced nephropathy
CTO: Chronic total occlusion
DA: Diagnostic angiography
DAPT: Dual antiplatelet therapy
GFR: Glomerular filtration rate
LM: Left main coronary artery
MACCE: Major adverse cardiovascular or cerebral event
MACE: Major adverse cardiovascular event
MV: Multivessel
ND: No data
n. s.: Not statistically significant (*p* < 0.05)
NS: Not specified
NSTEMI: Non-ST-segment elevation myocardial infarction
OBS: Observational study
OCT: Optical coherence tomography
OR: Odds ratio
OS: Overnight stay
PCI: Percutaneous coronary intervention
PCP: Percutaneous coronary procedures
RA: Rotational atherectomy
RCT: Randomized controlled trial
SDD: Same-day discharge
STEMI: ST-segment elevation myocardial infarction
SVG: Saphenous vein graft
TLR: Target lesion revascularization
TTE: Transthoracic echocardiography
TVR: Target vessel revascularization

## References

[1] Rao SV, Ou FS, Wang TY, Roe MT, Brindis R, Rumsfeld JS, Peterson ED (2008). Trends in the prevalence and outcomes of radial and femoral approaches to percutaneous coronary intervention: a report from the national cardiovascular data registry. JACC Cardiovasc Interv.

[2] Waldo SW, Gokhale M, O’Donnell CI, Plomondon ME, Valle JA, Armstrong EJ, Schofield R, Fihn SD, Maddox TM (2018). Temporal trends in coronary angiography and percutaneous coronary intervention: insights from the VA clinical assessment, reporting, and tracking program. JACC Cardiovasc Interv.

[3] Rao SV, Vidovich MI, Gilchrist IC, Gulati R, Gutierrez JA, Hess CN, Kaul P, Martinez SC, Rymer J (2021). 2021 ACC expert consensus decision pathway on same-day discharge after percutaneous coronary intervention: a report of the American college of cardiology solution set oversight committee. J Am Coll Cardiol.

[4] Box LC, Blankenship JC, Henry TD, Messenger JC, Cigarroa JE, Moussa ID, Snyder RW, Duffy PL, Carr JG, Tukaye DN (2020). SCAI position statement on the performance of percutaneous coronary intervention in ambulatory surgical centers. Catheter Cardiovasc Interv.

[5] Seto AH, Shroff A, Abu-Fadel M, Blankenship JC, Boudoulas KD, Cigarroa JE, Dehmer GJ, Feldman DN, Kolansky DM, Lata K (2018). Length of stay following percutaneous coronary intervention: an expert consensus document update from the society for cardiovascular angiography and interventions. Catheter Cardiovasc Interv.

[6] Heyde GS, Koch KT, de Winter RJ, Dijkgraaf MG, Klees MI, Dijksman LM, Piek JJ, Tijssen JG (2007). Randomized trial comparing same-day discharge with overnight hospital stay after percutaneous coronary intervention: results of the elective PCI in outpatient study (EPOS). Circulation.

[7] Ziakas A, Klinke P, Fretz E, Mildenberger R, Williams MB, Siega AD, Kinloch RD, Hilton JD (2004). Same-day discharge is preferred by the majority of the patients undergoing radial PCI. J Invasive Cardiol.

[8] Kim M, Muntner P, Sharma S, Choi JW, Stoler RC, Woodward M, Mann DM, Farkouh ME (2013). Assessing patient-reported outcomes and preferences for same-day discharge after percutaneous coronary intervention: results from a pilot randomized, controlled trial. Circ Cardiovasc Qual Outcomes.

[9] Perla RJ, Hohmann SF, Annis K (2013). Whole-patient measure of safety: using administrative data to assess the probability of highly undesirable events during hospitalization. J Healthc Qual.

[10] Zhang X, Hauck K, Zhao X (2013). Patient safety in hospitals—a Bayesian analysis of unobservable hospital and specialty level risk factors. Health Econ.

[11] Din JN, Snow TM, Rao SV, Klinke WP, Nadra IJ, Della Siega A, Robinson SD (2017). Variation in practice and concordance with guideline criteria for length of stay after elective percutaneous coronary intervention. Catheter Cardiovasc Interv.

[12] Januzzi JL, Ahmad T, Binder LG, Hucker WJ, Kumbhani DJ, Maddox TM, Marine JE, Morris PB (2019). 2019 Methodology for creating expert consensus decision pathways: a report of the American college of cardiology. J Am Coll Cardiol.

[13] Chambers CE, Dehmer GJ, Cox DA, Harrington RA, Babb JD, Popma JJ, Turco MA, Weiner BH, Tommaso CL, Society for Cardiovascular (2009). Defining the length of stay following percutaneous coronary intervention: an expert consensus document from the society for cardiovascular angiography and interventions. Endorsed by the American college of cardiology foundation. Catheter Cardiovasc Interv.

[14] Brandt MC, Alber H, Berger R, Binder RK, Mascherbauer J, Niessner A, Schmid M, Wernly B, Frick M, cardiology o. b. o. t. w. g. o. i. Same-day discharge after percutaneous coronary procedures—structured review and comprehensive meta-analysis. Wien Klin Wochenschr. 2024;. 10.1007/s00508-024-02347-z PMC11093862 38743083

[15] Block PC, Ockene I, Goldberg RJ, Butterly J, Block EH, Degon C, Beiser A, Colton T (1988). A prospective randomized trial of outpatient versus inpatient cardiac catheterization. N Engl J Med.

[16] Bradley SM, Kaltenbach LA, Xiang K, Amin AP, Hess PL, Maddox TM, Poulose A, Brilakis ES, Sorajja P, Ho PM, Rao SV (2021). Trends in use and outcomes of same-day discharge following elective percutaneous coronary intervention. JACC Cardiovasc Interv.

[17] Madan M, Bagai A, Overgaard CB, Fang J, Koh M, Cantor WJ, Garg P, Natarajan MK, So DYF, Ko DT (2019). Same-day discharge after elective percutaneous coronary interventions in Ontario, Canada. J Am Heart Assoc.

[18] Rubimbura V, Rostain L, Duval AM, Akakpo S, Boukantar M, Boiron P, Mouillet G, Gallet R, Belarbi A, Le Corvoisier P (2019). Outcomes and safety of same-day discharge after percutaneous coronary intervention: a 10-year single-center study. Catheter Cardiovasc Interv.

[19] Brayton KM, Patel VG, Stave C, de Lemos JA, Kumbhani DJ (2013). Same-day discharge after percutaneous coronary intervention: a meta-analysis. J Am Coll Cardiol.

[20] Abdelaal E, Rao SV, Gilchrist IC, Bernat I, Shroff A, Caputo R, Costerousse O, Pancholy SB, Bertrand OF (2013). Same-day discharge compared with overnight hospitalization after uncomplicated percutaneous coronary intervention: a systematic review and meta-analysis. JACC Cardiovasc Interv.

[21] Bundhun PK, Soogund MZ, Huang WQ (2017). Same day discharge versus overnight stay in the hospital following percutaneous coronary intervention in patients with stable coronary artery disease: a systematic review and meta-analysis of randomized controlled trials. Plos One.

[22] Lu H, Guan W, Zhou Y, Bao H (2019). Early versus late clinical outcomes following same day discharge after elective percutaneous coronary intervention: a systematic review and meta-analysis. Medicine.

[23] Bertrand OF, Rodes-Cabau J, Larose E, Nguyen CM, Roy L, Dery JP, Courtis J, Nault I, Poirier P, Costerousse O, De Larochelliere R (2008). One-year clinical outcome after abciximab bolus-only compared with abciximab bolus and 12-hour infusion in the randomized EArly discharge after transradial stenting of coronarY arteries (EASY) study. Am Heart J.

[24] Jabara R, Gadesam R, Pendyala L, Chronos N, Crisco LV, King SB, Chen JP (2008). Ambulatory discharge after transradial coronary intervention: preliminary US single-center experience (same-day transradial intervention and discharge evaluation, the STRIDE study). Am Heart J.

[25] Taxiarchi P, Kontopantelis E, Kinnaird T, Curzen N, Banning A, Ludman P, Shoaib A, Rashid M, Martin GP, Mamas MA (2020). Adoption of same day discharge following elective left main stem percutaneous coronary intervention. Int J Cardiol.

[26] Koutouzis M, Karatasakis A, Brilakis ES, Agelaki M, Maniotis C, Dimitriou P, Lazaris E (2017). Feasibility and safety of same-day discharge after complex percutaneous coronary intervention using forearm approach. Cardiovasc Revasc Med.

[27] Hodkinson EC, Ramsewak A, Murphy JC, Shand JA, McClelland AJ, Menown IB, Hanratty CG, Spence MS, Walsh SJ (2013). An audit of outcomes after same-day discharge post-PCI in acute coronary syndrome and elective patients. J Interv Cardiol.

[28] Small A, Klinke P, Siega DA, Fretz E, Kinloch D, Mildenberger R, Williams M, Hilton D (2007). Day procedure intervention is safe and complication free in higher risk patients undergoing transradial angioplasty and stenting. The discharge study. Catheter Cardiovasc Interv.

[29] Cordoba-Soriano JG, Rivera-Juarez A, Gutierrez-Diez A, Gutierrez-Ibanes E, Gallardo-Lopez A, Samaniego-Lampon B, Lozano I, Melehi D, Portero-Portaz JJ, Elizaga J, Jimenez-Mazuecos J (2019). The feasibility and safety of ambulatory percutaneous coronary interventions in complex lesions. Cardiovasc Revasc Med.

[30] Kok MM, Weernink MGM, von Birgelen C, Fens A, van der Heijden LC, van Til JA (2018). Patient preference for radial versus femoral vascular access for elective coronary procedures: the PREVAS study. Catheter Cardiovasc Interv.

[31] Cooper CJ, El-Shiekh RA, Cohen DJ, Blaesing L, Burket MW, Basu A, Moore JA (1999). Effect of transradial access on quality of life and cost of cardiac catheterization: a randomized comparison. Am Heart J.

[32] Perret X, Bergerot C, Rioufol G, Bonvini RF, Ovize M, Finet G (2009). Same-day-discharge ad hoc percutaneous coronary intervention: initial single-centre experience. Arch Cardiovasc Dis.

[33] Oh HL, Gwon HC, Lee SM, Kim YH, Cheon IS, Cheon WJ, Choi JH, Lee SC, Sung JD, Kim JS (2004). Safety of one-day admission transradial coronary intervention. Korean Circ J.

[34] Chen Y, Lin FF, Marshall AP (2021). Patient and family perceptions and experiences of same-day discharge following percutaneous coronary intervention and those kept overnight. Intensive Crit Care Nurs.

[35] Le Corvoisier P, Gellen B, Lesault P-F, Cohen R, Champagne S, Duval A-M, Montalescot G, Elhadad S, Montagne O, Durand-Zaleski I (2013). Ambulatory transradial percutaneous coronary intervention: a safe, effective, and cost-saving strategy. Cathet Cardio Intervent.

[36] Liew S, Dinh D, Liew D, Brennan A, Duffy S, Reid C, Lefkovits J, Stub D, Investigators V (2020). Prevalence, outcomes and cost implications of patients undergoing same day discharge after elective percutaneous coronary intervention in Australia. Heart Lung Circ.

[37] Amin AP, Pinto D, House JA, Rao SV, Spertus JA, Cohen MG, Pancholy S, Salisbury AC, Mamas MA, Frogge N (2018). Association of same-day discharge after elective percutaneous coronary intervention in the United States with costs and outcomes. JAMA Cardiol.

[38] Amin AP, Crimmins-Reda P, Miller S, Rahn B, Caruso M, Pierce A, Dennis B, Pendegraft M, Sorensen K, Kurz HI, et al. Novel patient-centered approach to facilitate same-day discharge in patients undergoing elective percutaneous coronary intervention. J Am Heart Assoc. 2018;7(4). 10.1161/JAHA.117.005733 PMC5850176 29449273

[39] Kopin D, Seth M, Sukul D, Dixon S, Aronow HD, Lee D, Tucciarone M, Pielsticker E, Gurm HS (2019). Primary and secondary vascular access site complications associated with percutaneous coronary intervention: insights from the BMC2 registry. JACC Cardiovasc Interv.

[40] Dietz U, Holz N, Dauer C, Lambertz H (2006). Shortening the stent length reduces restenosis with bare metal stents: matched pair comparison of short stenting and conventional stenting. Heart.

[41] Shirai S, Kimura T, Nobuyoshi M, Morimoto T, Ando K, Soga Y, Yamaji K, Kondo K, Sakai K, Arita T (2010). Impact of multiple and long sirolimus-eluting stent implantation on 3-year clinical outcomes in the j-cypher registry. JACC Cardiovasc Interv.

[42] Konishi H, Miyauchi K, Dohi T, Tsuboi S, Ogita M, Naito R, Kasai T, Tamura H, Okazaki S, Isoda K, Daida H (2016). Impact of stent length on clinical outcomes of first-generation and new-generation drug-eluting stents. Cardiovasc Interv Ther.

[43] Chang SH, Chen CC, Hsieh MJ, Wang CY, Lee CH, Hsieh IC (2013). Lesion length impacts long term outcomes of drug-eluting stents and bare metal stents differently. Plos One.

[44] Chandrasekhar J, Baber U, Sartori S, Stefanini GG, Sarin M, Vogel B, Farhan S, Camenzind E, Leon MB, Stone GW (2018). Effect of increasing stent length on 3-year clinical outcomes in women undergoing percutaneous coronary intervention with new-generation drug-eluting stents: patient-level pooled analysis of randomized trials from the WIN-DES initiative. JACC Cardiovasc Interv.

[45] Ali ZA, Maehara A, Genereux P, Shlofmitz RA, Fabbiocchi F, Nazif TM, Guagliumi G, Meraj PM, Alfonso F, Samady H (2016). Optical coherence tomography compared with intravascular ultrasound and with angiography to guide coronary stent implantation (ILUMIEN III: OPTIMIZE PCI): a randomised controlled trial. Lancet.

[46] Chamie D, Bezerra HG, Attizzani GF, Yamamoto H, Kanaya T, Stefano GT, Fujino Y, Mehanna E, Wang W, Abdul-Aziz A (2013). Incidence, predictors, morphological characteristics, and clinical outcomes of stent edge dissections detected by optical coherence tomography. JACC Cardiovasc Interv.

[47] Ali ZA, Karimi Galougahi K, Mintz GS, Maehara A, Shlofmitz RA, Mattesini A (2021). Intracoronary optical coherence tomography: state of the art and future directions. EuroIntervention.

[48] Ali ZA, Karimi Galougahi K, Maehara A, Shlofmitz RA, Ben-Yehuda O, Mintz GS, Stone GW (2017). Intracoronary optical coherence tomography 2018: current status and future directions. JACC Cardiovasc Interv.

[49] Mehran R, Nikolsky E (2006). Contrast-induced nephropathy: definition, epidemiology, and patients at risk. Kidney Int Suppl.

[50] Gurm HS, Dixon SR, Smith DE, Share D, LaLonde T, Greenbaum A, Moscucci M (2011). Renal function-based contrast dosing to define safe limits of radiographic contrast media in patients undergoing percutaneous coronary interventions. J Am Coll Cardiol.

[51] Jang JS, Jin HY, Seo JS, Yang TH, Kim DK, Kim DK, Kim DI, Cho KI, Kim BH, Park YH (2012). The transradial versus the transfemoral approach for primary percutaneous coronary intervention in patients with acute myocardial infarction: a systematic review and meta-analysis. EuroIntervention.

[52] Ferrante G, Rao SV, Juni P, Da Costa BR, Reimers B, Condorelli G, Anzuini A, Jolly SS, Bertrand OF, Krucoff MW (2016). Radial versus femoral access for coronary interventions across the entire spectrum of patients with coronary artery disease: a meta-analysis of randomized trials. JACC Cardiovasc Interv.

[53] Ndrepepa G, Colleran R, Braun S, Cassese S, Hieber J, Fusaro M, Kufner S, Ott I, Byrne RA, Husser O (2016). High-sensitivity troponin T and mortality after elective percutaneous coronary intervention. J Am Coll Cardiol.

[54] Prasad A, Rihal CS, Lennon RJ, Singh M, Jaffe AS, Holmes DR (2008). Significance of periprocedural myonecrosis on outcomes after percutaneous coronary intervention: an analysis of preintervention and postintervention troponin T levels in 5487 patients. Circ Cardiovasc Interv.

[55] Saad Y, Shugman IM, Kumar M, Pauk I, Mussap C, Hopkins AP, Rajaratnam R, Lo S, Juergens CP, French JK (2015). Safety and efficacy of same-day discharge following elective percutaneous coronary intervention, including evaluation of next day troponin T levels. Heart Lung Circ.

[56] Mols RE, Hald M, Vistisen HS, Lomborg K, Maeng M (2019). Nurse-led motivational telephone follow-up after same-day percutaneous coronary intervention reduces readmission and contacts to general practice. J Cardiovasc Nurs.

[57] Kok MM, von Birgelen C, Lam MK, Lowik MM, van Houwelingen KG, Stoel MG, Louwerenburg JH, de Man FH, Hartmann M, Doggen CJ (2016). Patient preference regarding assessment of clinical follow-up after percutaneous coronary intervention: the PAPAYA study. EuroIntervention.

[58] Koutouzis M, Liontou C, Xenogiannis I, Tajti P, Tsiafoutis I, Lazaris E, Oikonomidis N, Kontopodis E, Rangan B, Brilakis E (2021). Same day discharge after chronic total occlusion interventions: a single center experience. Catheter Cardiovasc Interv.

[59] Gaba P, Serruys PW, Karmpaliotis D, Lembo NJ, Banning AP, Zhang Z, Morice MC, Kandzari DE, Gershlick AH, Ben-Yehuda O (2021). Outpatient versus inpatient percutaneous coronary intervention in patients with left main disease (from the EXCEL trial). Am J Cardiol.

[60] Abdel-Razek O, Jung Y, Jung R, Skanes S, Dhaliwal S, Stotts C, Di Santo P, Goh CY, Verreault-Julien L, Visintini S (2022). Safety of same-day discharge in patients with left main percutaneous intervention. Coron Artery Dis.

[61] Gilchrist IC, Rhodes DA, Zimmerman HE (2012). A single center experience with same-day transradial-PCI patients: a contrast with published guidelines. Catheter Cardiovasc Interv.

